# Epigenetic Modification Factors and microRNAs Network Associated with Differentiation of Embryonic Stem Cells and Induced Pluripotent Stem Cells toward Cardiomyocytes: A Review

**DOI:** 10.3390/life13020569

**Published:** 2023-02-17

**Authors:** Afshin Zare, Aria Salehpour, Arezoo Khoradmehr, Shabnam Bakhshalizadeh, Vahid Najafzadeh, Sahar Almasi-Turk, Mahdi Mahdipour, Reza Shirazi, Amin Tamadon

**Affiliations:** 1 The Persian Gulf Marine Biotechnology Research Center, The Persian Gulf Biomedical Sciences Research Institute, Bushehr University of Medical Sciences, Bushehr 7514633196, Iran; 2 Reproductive Development, Murdoch Children’s Research Institute, Melbourne, VIC 3052, Australia; 3 Department of Paediatrics, University of Melbourne, Melbourne, VIC 3010, Australia; 4 Department of Veterinary and Animal Sciences, University of Copenhagen, 1870 Frederiksberg C, Denmark; 5 Department of Basic Sciences, School of Medicine, Bushehr University of Medical Sciences, Bushehr 7514633341, Iran; 6 Stem Cell Research Center, Tabriz University of Medical Sciences, Tabriz 5166653431, Iran; 7 Department of Reproductive Biology, Faculty of Advanced Medical Sciences, Tabriz University of Medical Sciences, Tabriz 5166653431, Iran; 8 Department of Anatomy, School of Medical Sciences, Medicine & Health, UNSW Sydney, Sydney, NSW 2052, Australia; 9 PerciaVista R&D Co., Shiraz 7135644144, Iran

**Keywords:** epigenetic markers, cardiomyocyte, proliferation, differentiation, induced pluripotent stem cell, embryonic stem cell

## Abstract

More research is being conducted on myocardial cell treatments utilizing stem cell lines that can develop into cardiomyocytes. All of the forms of cardiac illnesses have shown to be quite amenable to treatments using embryonic (ESCs) and induced pluripotent stem cells (iPSCs). In the present study, we reviewed the differentiation of these cell types into cardiomyocytes from an epigenetic standpoint. We also provided a miRNA network that is devoted to the epigenetic commitment of stem cells toward cardiomyocyte cells and related diseases, such as congenital heart defects, comprehensively. Histone acetylation, methylation, DNA alterations, N6-methyladenosine (m^6^a) RNA methylation, and cardiac mitochondrial mutations are explored as potential tools for precise stem cell differentiation.

## 1. Introduction

The American Heart Association reported a 363.4-billion-dollar cost for the United States’ cardiovascular diseases, recently [1]. Despite major advancements in the treatment of various cardiac disorders, there being rising mortality rates [2] and a greater burden on healthcare expenditures [3] are anticipated. Some people have described these undesired statistics as the inertia of cardiovascular disease therapy, and therefore, more advanced therapies are being demanded [4]. To comprehend the treatment challenges, we should take a look at heart contraction-producing cells called “cardiomyocytes,” which are inherently different at various stages of maturation. At the adult stage, they reveal characteristics such as longer sarcomeres (2.2 vs. 1.65 μm), lower resting membrane potential, and a dependence on oxidative phosphorylation instead of glycolysis [5,6]. One major characteristic of adult cardiomyocytes which causes problems with the treatment is terminal differentiation. So, after birth, the proliferation is replaced with hypertrophy in the cardiomyocytes. The terminal differentiation phenomenon is executed via either the upregulation of cardiac-specific adult genes or the termination of cardiac cell division [7,8,9]. Additionally, epigenetic mechanisms have been shown to contribute to the occurrence of terminal differentiation [7].

One of the suggested treatments after an infarction is the rejuvenation of the myocardial tissue. To this end, researchers have proposed various sources of stem cells with the capability of differentiating into cardiomyocytes. Three distinct categories may be used to group these cell types. (I) The “First-generation stem cells” typically include the mesenchymal stem cells (MSCs) [10], the hematopoietic stem cells, the endothelial progenitor cells, and the mononuclear cells [11], which are stem cells produced from adult tissues and bone marrow. The “Second-generation stem cells” are committed stem cells that are similar to pluripotent stem cells [12] and induced pluripotent stem cells (iPSCs), patches, exosomes, and they are derivatives of Wharton’s jelly which make up the “third generation of stem cell treatment” [13].

An optimal stem cell candidate for the cell treatment of cardiac disorders in regenerative medicine should meet two important requirements: (I) they should not trigger immunological responses, and (II) they should be being capable of differentiating into the desired cardiovascular cells. One of the pluripotent stem cell resources which are known to be capable of differentiating into cardiomyocytes are human embryonic stem cells (ESCs) [14]. However, their immunogenic potential and ethical constraints are the main objections when one is considering these cell lines [15]. In contrast, researchers have focused on personalized iPSCs since they can be produced by the same person by the nuclear reprogramming of their somatic cells, thus, they are an alternative source for cardiomyocyte production [16]. It has been well documented that the direct transplantation of stem cells into the heart tissue via injection could result in teratoma. Therefore, before their implantation, the effective differentiation of the stem cells into cardiomyocytes is essential [17].

There are now three basic methods for differentiating pluripotent stem cells into cardiomyocytes. The first technique involves co-culturing the pluripotent stem cells with the stromal cells that resemble mouse visceral endoderm (END-2) or in END-2-conditioned media [18]. Additionally, it has been demonstrated that MAPK inhibitors can improve the efficiency of Stem cell differentiation to cardiomyocyte yield. [19]. The second technique for differentiating pluripotent stem cells into cardiomyocytes uses embryoid bodies in suspension. The pluripotent stem cells begin to differentiate into cardiomyocytes when a set of chemical and physical conditions that simulate early embryonic development are present. The pluripotent stem cells gather to form embryoid bodies, which are then co-cultured for a prolonged period, causing 5–70% of the embryoid bodies to differentiate into cardiomyocytes. However, this approach has numerous limitations, including a disparity in the number of beating cardiomyocytes across the embryoid bodies, a poor yield efficiency, and an immature cardiomyocyte phenotype [20].

The last proposed method is two-dimensional monolayer differentiation which is based on adding small molecules and growth factors to the culture medium [21]. This approach results in the successful differentiation of the majority of the cells (85–95%), and subsequently, it yields more mature cardiomyocytes, [22].

Cell nuclear reprogramming via the epigenetic modifications of the cells has a complex mechanism and is conducted via the regulation of the chromatin structure [23]. Furthermore, this mechanism is performed to provide the conditions for the specialization and differentiation of various cell types [24,25]. In the iPSCs, the epigenetics and chromatin modifications are critical but reversible dynamics [26]. Numerous epigenetic factors cooperate with the transcription factors and the signaling pathways to facilitate the cardiomyocyte differentiation process. These epigenetic factors encompass DNA methylation, ATP-dependent chromatin remodeling complexes, and histone modifications [27]. By altering the availability of the DNA sequences for DNA-binding proteins, the epigenetic regulators may affect the expression or repression of the genes. These changes result in looser or tighter chromatin via the altering of the DNA-histone covalent bonds, respectively. As a result, the DNA will either become more or less accessible, respectively. The major focus of this review is the epigenetic regulation of the ESCs and iPSCs into cardiomyocytes.

Another field of interest in promoting regeneration in the cardiac tissue are miRNAs. MiRNAs are basically 19–24 nucleotide RNAs that are encoded endogenously [28], and they downregulate the target mRNA by binding to the 3′-UTR region [29]. MiRNAs promote mRNA breakdown or impede mRNA translation to negatively control the gene expression at the post-transcriptional stage. More than 2600 mature miRNAs have been found in human cells so far [30], and it is known that these miRNAs use intricate methods to control how one-third of the human genes are expressed [31].

MiRNAs are essential controllers of myocardial development and disorders as well [32]. For instance, it has been shown that in vitro myocardial differentiation is promoted by upregulating the cardiac-abundant miRNAs in the ESCs, including mir-1 and mir-499 [33]. Additionally, it was shown that overexpressing miRNA-1 in ESCs improved the cardiac differentiation following a transplantation. Additionally, miRNA-1 overexpression in ESCs may have a positive paracrine impact by preventing cardiomyocytes from apoptosis in vivo [34,35].

## 2. Embryonic Stem Cells (ESCs)

The ESCs are considered to be an effective cell resource in regenerative medicine due to their inherent plasticity [36,37]. Pluripotent stem cells may be created utilizing a patient’s nuclear genome by cell nuclear transfer during animal cloning using ESCs. These cells may develop into cardiomyocytes, and ultimately, they may be employed to repair heart disease. Although this technology has been applied in animal models [38,39], it comes with some undesirable outcomes, which should be taken into consideration. The low efficiency and low pluripotency in the produced lines, the high demand for superovulation, the abnormalities of cloned cells, and there being an ethical debate for human oocyte manipulations are the main objections.

## 3. Induced Pluripotent Stem Cells (iPSCs)

Several protocols have been documented for somatic cell reprogramming to differentiate them into iPSCs. These procedures involve employing cell and transgene-free ESC protein extracts, delivering reprogramming factor transgenes by employing adenovirus vectors, lentivirus vectors, Sendai virus vectors, the transduction of Oct4, Sox2, Klf4, and c-Myc by retrovirus [16,40,41,42], the transfection of plasmids without the use of c-Myc [43], and by utilizing the PiggyBac system which is accompanied by a recombinant protein such as tetracycline transactivator (rtTA) [44,45,46,47,48].

The iPSCs are capable of generating all of the three germ lineages and since they contain autologous sources, they could have a syngeneic nature. However, as previously mentioned, the low efficiency of the derived iPSCs, the lengthy treatment procedures, and the propensity for teratoma formation are considered to be the disadvantages of using iPSCs [49]. The epigenetic mechanisms governing the production of iPSCs have not been fully elucidated yet. Therefore, understanding the underlying mechanisms of epigenetic reprogramming and their sustainability following reprogramming is crucial before applying them in a clinical setting.

ESCs and iPSCs are epigenetically highly complicated [50]. A pluripotency status is guaranteed when continuous proliferation and de-differentiation are accompanied by the ability to differentiate into a particular cell lineage. To consider a cell as being pluripotent, three fundamental criteria must be met: (i) it’s epigenetic factors should continuously and actively remain in a flexible balance; (ii) the pluripotency transcripts should remain upregulated, while their differentiating counterparts should be downregulated; (iii) the proper and in-time de-condensation of the chromatin sequences, which are responsible for the initiation of differentiating machinery in the cell [51,52,53,54,55,56,57,58,59]. In other words, for sustaining pluripotency in the ESCs and the iPSCs, a dynamic balance should exist between at least three epigenetic elements, including DNA methylation, chromatin structure, and microRNAs. Furthermore, these elements need to operate in conjunction with the transcription factors to sustain pluripotency [60].

## 4. Epigenetics of Gene Expression and Silencing

**Figure 1:**
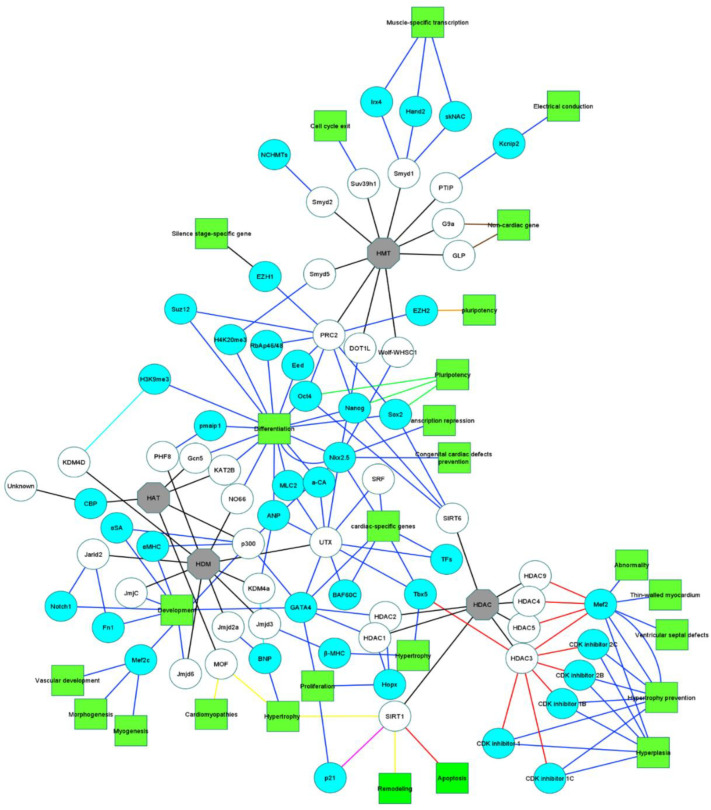
**Histone modifiers network and their effect on heart development genes and their function.** Histone acetyltransferases (HAT), histone deacetylases (HDAC), histone demethylase (HDM), and histone methyltransferase (HMT) (gray octagons) are four families that include (black line) different enzymes. Some of these enzymes (white circles) modify histones and effect cardiac evolution by impacting cardiac genes (light blue circles). Activation (dark blue line), inhibition (red line), deacetylation (purple line), up-regulation (light blue line), down-regulation (yellow line), silencing (dark brown line), execution (orange line), and maintaining (green line) are some of these modifications that are performed by these enzymes on cardiac genes. The results of the influence of these enzymes (white circles) on cardiac genes (light blue circles) are shown in Figure 1 as green triangles. (Figure 1 was generated using Cytoscape application—cytoscape.org (accessed on 28 September 2022)).

By definition, epigenetic alterations modify the gene expression without changing the genetic sequence. These modifications are carried out by a variety of mechanisms, such as post-transcriptional modifications (siRNA, miRNA, and piRNA), DNA methylation, modifications in the ATP-dependent chromatin remodeling complexes, and modifications to the histone proteins (acetylation, methylation, sumoylation, phosphorylation, deamination, ribosylation, and proline isomerization) (Table 1) [24,61,62]. The process of cardiac differentiation has been shown to involve the activation of cell cycle inhibitors, cardiac-specific genes, and the inhibition of cell cycle progression and non-cardiac transcripts (Figure 1) [63,64].

### 4.1. Histone Modifications

Firstly, we have to take a closer look to see where histones are located, basically. To this end, we have to become familiar with the nucleosomes. The nucleosomes are the repeating units of chromatin in which two superhelical strands of DNA twist around eight core histone proteins, which consist of an H3-H4 tetramer and two H2A-H2B dimers [65]. The N- and C-terminals of the histones are the sites for acetylation, methylation, ubiquitination, sumoylation, and phosphorylation, which histones are modified by, thus, influencing the gene transcription, epigenetically [66]. All of the relevant published information related to the histone modifications of stem cells for differentiation towards cardiomyocyte cells has been categorized in a more detailed manner.

#### 4.1.1. Histone Acetylation of Stem Cells toward Cardiomyocyte Differentiation

The acetylation of histones was first coined by Phillips [67]. Histone acetyltransferases (HATs) and histone deacetylases have competing roles in the dynamic regulation of the acetylation of histones (HDACs) [68]. The acetylation by HATs weakens the positive charge of a lysine residue and binds the histones and DNA, which leads to the additional exposure of the DNA. Therefore, acetylation is regarded as an activation marker for transcription [69].

P300, which is a HAT that suppresses the activity of the HDACs [70] and a transcriptional coactivator that interacts with: (i) GATA4 (which functions in several ways throughout the development of the heart) [71], (ii) myocyte-specific enhancer factor 2C (Mef2c), which is essential for cardiac morphogenesis, myogenesis, and angiogenesis [72], and (iii) Nkx-2.5 (which are important genes for controlling tissue differentiation and dictating the patterns of temporal and spatial development) [73]. Furthermore, p300 increases the expression of α-actinin and myosin [74]. P300 histone acetyltransferase activity contributes to embryonic myocardium development [75], and it is necessary for heart tissue regeneration as well [76].

The males absent on the first (MOF) protein acts as a HAT, which limits both mouse cardiac hypertrophy and human cardiomyopathy [77]. Another member of the HAT family is the cAMP response element-binding protein (CBP). Nonetheless, its absence does not affect the heart’s formation [78,79]. The other HAT, which is known as Gcn5, is involved in cardiac differentiation by altering the acetylation levels of H3 [80].

#### 4.1.2. Histone Deacetylation of Stem Cells toward Cardiomyocyte Differentiation

It has been revealed that the cardiac-specific simultaneous ablation of the HDAC1 and HDAC2 genes is accompanied by several serious consequences, including dilated cardiomyopathy, cardiac arrhythmias, and neonatal lethality, while the individual deletion of each gene does not result in the development of an abnormal specific phenotype [81]. HDAC2 in combination with Hopx (homeodomain-only protein), restricts the cardiomyocytes’ proliferation via two processes of GATA4 deacetylation and reduces its transcriptional activity [82,83]. 

HDAC3, which is another class I HDAC, was shown to cause the thickening of the ventricular myocardium while it was overexpressed [84], and the deletion of HDAC3 was correlated with a hypertrophic effect on the cardiac cells in the mice as well [85]. HDAC3 has suppression effects on the Tbx5 activity by binding at a subset of the Tbx5-bound sites [86], which plays an indispensable role in the early life stages of cardiac development [87]. Additionally, an increase in the level of cardiac hyperplasia through the suppression of cyclin-dependent kinase inhibitors such as (p21^cip1^), (p27^Kip1^), (p57^kip2^), (p18^inc4c^), and (p15^inc4b^) without hypertrophy is one of the main results for the overexpression of HDAC3 [84,87]. 

One of the HDACs which prevents cardiac hypertrophy is HDAC4, which suppresses Mef2 and regulates cardiac hypertrophy [88,89]. The functions of HDAC5 and HDAC9 are complementary during the heart’s development via Mef2 suppression. However, impairments in their function are connected to cardiomyocyte abnormalities, thin-walled myocardium, and ventricular septal defects [90,91].

The HDACs are activated by binding the Hey proteins proximally to the transcription starting sites which leads to the suppression of gene expression by the deacetylation of histones and nuclear condensation. In cardiac myocytes, the binding of cardiac inducers activates the histone acetylases, and hence, it inactivates the Hey proteins as a consequence [92].

In humans, there are seven identified sirtuins (SIRTs) that regulate the cellular activity. SIRT1, SIRT6, and SIRT7 are mostly found in the nucleus, SIRT2 is mostly found in the cytoplasm, and it is also translocated in the nucleoplasm, and SIRT3, SIRT4, and SIRT5 are mostly found in the mitochondria [93]. SIRT1, SIRT2, SIRT6, and SIRT7 of the seven mammalian sirtuins have been shown to have key epigenetic functions [94].

Previous research has shown how the Sirt1/PGC1/pAkt axis protects the cells from oxidative stress and senescence, which may be beneficial when one is employing MSCs [95,96]. A study has revealed that Resveratrol may lessen the oxidative stress and boost the survival pathways for adipose-derived MSCs during the high glucose conditioning of the cells by upregulating the Sirt1/PGC1/pAkt axis [97]. Furthermore, pre-exposing MSCs to glucose deprivation conditions may postpone senescence, increase proliferation, and restore the ability of the aged cells to heal the senescent-infarcted myocardium [98].

Doxorubicin is a powerful antitumor anthracycline antibiotic that is routinely used to treat a wide range of tumors. However, Doxorubicin’s medical usage is restricted due to its significant cardiotoxicity, which frequently results in permanent progressive cardiomyopathy and cardiac arrest [99]. Left ventricular dysfunction represents the most prevalent consequence of Doxorubicin cardiotoxicity [100]. In a study, it was revealed that the exosomes alleviated the Doxorubicin-derived cardiomyocyte degeneration, and these positive outcomes were conveyed via the putative exosome/lncRNA-NEAT1/miR-221-3p/Sirt2 pathway [101]. Another study also pointed out that LncRNA KLF3-AS1 in the exosomes released from the MSCs may control Sirt1 to prevent cell pyroptosis and slow the development of myocardial infarction [102]. 

Another member of the SIRTs family, SIRT3, has a beneficial impact on cellular aging and expectancy, and it can enhance the differentiation of MSCs as well [103,104]. According to research, human MSCs become less resistant to damage as they age. The upregulation of SIRT3 may shield the MSCs from oxidative degradation by moving FOXO3a into the cell nucleus, which activates catalase and manganese superoxide dismutase [105].

Persistent AMPK activation enhances the iPSC-derived myocytes differentiation, as demonstrated by the GLUT4 and CD36 membrane recruitment and the enhanced mitochondrial function. Additionally, AMPK upregulation raises NAD/NADH levels, which might be used by SIRT1, SIRT2, and SIRT6 to reduce the lysine acetylation [106].

The acetylation of H3K56 corresponds with the increased expression of the pluripotency-associated loci in ESCs [107]. SIRT6 is a NAD-dependent histone deacetylase that addresses acetylated H3K56 in mouse ESCs [108]. One of the significant roles of SIRT6 is the expression regulation of the pluripotency-inducing genes such as Oct4, Sox2, and Nanog via the deacetylation of H3K56. This results in ESC differentiation through the Tet-mediated oxidation of 5 mC into 5hmC [109].

In another approach, hMSCs were cultivated on polycaprolactone platforms that were three-dimensionally coordinated. Based on the upregulation of the cardiovascular indicators and intracellular calcium permeability, the collagen-covered filaments were shown to be the most efficient in inducing cardiomyogenesis among the various toppings. Increased SIRT6 levels were linked to the improved differentiation on the collagen-covered filaments at the cellular scale. By triggering the Wnt signaling pathway, the siRNA-mediated SIRT6 deficiency slowed the differentiation [110]. 

#### 4.1.3. Histone Methylation of Stem Cells toward Cardiomyocyte Differentiation

It was first documented that methionine is the methyl donor for N-methyl lysine in histones [111]. The variety of amino acids and mono-, di-, or trimethylation of the amino acid residues in the histones are two major elements that regulate the transcription activation. Methylation is one of the key post-transcriptional changes that happens at the H3 and H4 histones, as well as on the side chains of lysines and arginines [112,113]. For instance, the H4K20me1, H2BK5me1, and H3K36me3-enriched regions of chromatin are considered as active markers of transcription [114], while the promoters with H3K27me3 or H3K9me3 enrichment are considered to be inactive-associated regions of chromatin [51]. Generally, the transcription factors have more access to the loosened euchromatin structure, while the condensed heterochromatin structure prevents the transcription factors from accessing the genes of interest [115].

Histone methylation plays a crucial role in modifying cardiac evolution [116,117,118,119,120,121,122]. De novo mutations in the genes that alter H3K4 and H3K27 cause congenital cardiac abnormalities [123]. PAX-interacting protein 1 (PTIP), which is known as the H3K4 histone methyltransferase component, is important for altering the expression of the genes that assist in cardiac electrical conduction, notably, Kv channel-interacting protein 2 (Kcnip2) [124]. In the cardiac cells with PTIP having been knocked out, salt and calcium handle the dysregulations that take place [125], but no change happens during cardiac growth [124]. H3K4 histone methyltransferase does not affect the genes for myosin heavy chain beta (MHC-beta) and atrial natriuretic peptide (ANP), which are thought to be the cardiac hypertrophy genes [124].

Smyd1, which is a muscle-specific gene activator, methylates H3K4 and increases the levels of the Iroquois homeobox 4 and the heart and neural crest derivatives-expressed protein 2 (Hand2) (Irx4). Through the methylation of H3K4 and the overexpression of Hand2 and Irx4, Smyd1 may interact with the skeletal nascent polypeptide-associated complex alpha (skNAC). The right ventricle develops as a result of this contact, which also causes cardiomyocyte maturation [117,126,127,128,129]. It is yet unclear how directly suppressing Symd1 affects the activity of histone methyltransferase during the formation of the heart [108,111]. Symd1 is crucial for sarcomere organization by binding to myosin protein (which is known as the basic contractile unit of muscle) [127,130,131]. A neonatal cardiomyocyte histone methyltransferase of H3K4 and H3K36 is known as Smyd2. Interestingly, if Smyd2 is deficient, the other histone methyltransferases, such as Smyd1, can compensate for it [132,133].

Wolf-WHSC1, which is another histone methyltransferase, may mono-, di-, or trimethylate H3K36, which silences WHSC1. This leads to a congenital cardiac defect that is known as Wolf–Hirschhorn Syndrome, consequently [134]. The patients with WHSC1 deficiency have ventricular and atrial septal defects-related signs and symptoms [116]. Transcription is repressed when the Nkx2.5 target genes are occupied by WHSC1 via the trimethylation of H3K36 [120]. 

Additionally, Polycomb repressive complex 2 (PRC2) is a histone methyltransferase compound that occupies the Oct4, Sox2, and Nanog areas to activate the target genes and preserve pluripotency throughout the differentiation of the ESCs [53]. The PRC2 complex is the only identified methyltransferase of H3K27 in mouse ESCs [135] It comprises four main subsets: embryonic ectoderm development (Eed), the retinoblastoma protein-associated protein 46/48 (RbAp46/48), the catalytic subunit enhancer of Zeste 1 Ezh1/Ezh2, and the Zeste 12 (Suz12) suppressor [136].

The Ezh1 subset mediates the methylation on histone H3K27, complements the Ezh2 function, and plays a critical role in maintaining stem cell pluripotency [137]. On the other hand, Suz12 acts toward the differentiation of the stem cells, and its ablation is correlated with impaired activation of the downstream differentiation genes [138]. Contrary to Ezh2 and Eed, Eed is important for H3K27 di, tri, and monomethylation [139]. Additionally, PRC2 contributes significantly to the heart’s development.

The genes involved in the development of cardiac disease are significantly influenced by Ezh2 in terms of how they are expressed. When cardiac differentiation occurs, the Six1 transcription factor, which is active in the stem cell state, is silenced by Ezh2 [140,141]. The inactivated X chromosomes in female mammalian somatic cells have a high level of methylation H3K27 [142]. An increase in trimethylated H3K27 triggers PRC2’s steady recruitment, which causes cardiac progenitor cells to differentiate into cardiomyocytes [140]. In the yeast, *S. cerevisiae*, trimethylated H3K4 completely activates the promoter region of the genes (such as r1), while H3K4 demethylation enables transcription [143]. Before the transcriptional activation, the acquisition of trimethylated H3K4, and the recruitment of phosphorylated RNA polymerase II at serine 5, a monomethylated H3K4 pattern is present at the transcription sites of the genes (RNAP). This pattern of pre-activation is critical for the genes that are not regulated by the polycomb complexes [64]. The expression of Ezh1 and Ezh2 in adult and embryonic hearts is predominant, and this condition results in ceasing cardiac differentiation by gene silencing [140,141,144] [141,142,145]. Additionally, Ezh2 attaches to GATA4 and methylates it. This action has three main consequences: (i) deducting in the interaction between GATA4 and p300, (ii) the reduction in the transcriptional activity of GATA4, and (iii) the inhibition of the expression of the myosin heavy chain in fetal heart cells [145].

Moreover, the methyltransferases, G9a and GLP, silence the genes during differentiation by mono- and dimethylating H3K9 in the cardiomyocytes [146]. The G zero phase outset controls the cardiac differentiation process via the trimethylation of H3K9. Suv39h1 is involved in a quiescent state induction. To reduce the transcriptional activity, the suppressive markers such as di- and trimethylated H3K9 and trimethylated H3K27 are used. In the end, this suppression results in non-cardiac gene suppression and cell cycle halt [144].

There have also been reports of other crucial elements for the development of induced pluripotent stem cells into cardiomyocytes. Some of these supposedly essential components are tiny compounds that are used to control the epigenetic regulators. One of these is the histone methyltransferase inhibitor, BIX01294 [147], RG108 and 5-azacytidine, the DNA methyltransferase inhibitors [148,149], and valproic acid, which is a histone deacetylase inhibitor [150].

#### 4.1.4. Histone Demethylation of Stem Cells toward Cardiomyocyte Differentiation 

Jumonji proteins are essential for the growth of the cardiomyocytes [151]. A DNA-binding domain, an AT-rich interaction domain (ARID), and two conserved domains are found in the protein jumonji, which is encoded by the Jarid2 gene (JmjN and JmjC) [152]. Histone demethylation requires the JmjC domain [152,153]. Another member of this family, Jmjd6, functions as an H3 and H4 arginine histone demethylase. Studies have shown that Jmjd6 is also important in the formation of cardiac cells [154]. The trimethylated histones, H3K9 and H3K36, are demethylated by JMJD2A [155]. JMJD2A is a class of histone trimethyl demethylase that has been shown to become upregulated, while cardiac hypertrophy and long-term pressure overload happen. More accurately, in response to contractility, it is concentrated in the promoters of the atrial natriuretic peptide (ANP) and the brain natriuretic peptide (BNP) [89,156].

The ubiquitously transcribed tetratricopeptide repeat, X chromosome (UTX), an H3K27 demethylase, is the name of the second JmjC protein. The process of encoding UTX occurs on the X chromosome [157,158,159]. Cardiomyocyte development is reported to be affected following the up-regulation of UTX, which is necessary for cardiomyocyte development [102]. UTX influences the cardiomyocyte development by many methods, which are as follows: (i) by affecting the process of ESC differentiation into cardiomyocytes, (ii) by influencing the expression of cardiac-specific genes (ANP, MLC2, and a-CA) the through demethylation of trimethylated H3K27, (iii) by interacting with the various factors involved in organogenesis, such as TFs, Nkx2.5, Tbx5, GATA4, the serum response factor (SRF), and the Brg1-associated factor Baf60c (which results in the activation of cardiac-specific genes), (iv) by the demethylation of H3K4, which leads to the activation of the cardiac enhancers [122].

The aforementioned epigenetic histone markers and their impact on various cardiac-specific genes, the result of their influence on the cardiac tissue, and the utilized laboratory model are comprehensively gathered in Table 1.

### 4.2. Methylations

#### 4.2.1. DNA Methylation

Although methylation is not required for the ESCs to retain their stem cell identity, it is required for the differentiation to begin [160]. The enzymes called DNA methyltransferase (dnmt1, dnmt3a, and dnmt3b) attach a methyl group to the 50-CpG-30 dinucleotides. DNA methylation takes place on the fifth carbon position of the cytosine nucleotide. When a CG dinucleotide is formed, it leads to the repression of chromatin and the inhibition of the gene expression [161]. DNA methylation might perform a variety of activities, including X-chromosome inactivation, cell differentiation, chromatin structural modifications, cancer, genomic imprinting, tissue-specific gene expression, and pluripotency in the somatic cells [161,162]. The demethylation of the genes, however, could induce pluripotency in the somatic cells via nuclear reprogramming, thus generating iPSCs. AID, which is a cytidine deaminase molecule, plays an important part in either somatic hypermutation (SHM) or class-switch recombination (CSR) during reprogramming [163]. DNA methylation is catalyzed by factors including TET enzymes (TET1, TET2, and TET3), Fe^2+^- and α-ketoglutarate-dependent dioxygenases [164,165].

5-formylcytosine (5fC), 5-hydroxymethylcytosine (5 hmC), and 5-carboxycytosine (5caC) are intermediates in the mechanism underlying active DNA demethylation [166,167]. These three intermediary factors are produced via 5-methylcytosine (5 mC) oxidation, which is catalyzed by the TET enzymes, affecting the methylation status of the DNA [166,167]. In order to sustain pluripotency, two important factors should increase: (i) the level of the expression of the genes that are associated with TET1 and TET2, and (ii) the oxidation of 5-methylcytosine (5 mC) to produce 5 hmC [168]. It has been revealed that during the ESC culture, the amount of 5 hmC decreases in the first four days, and then, it increases in the following four days. Simultaneously, Tet1 showed constant downregulation, and Tet3 showed constant upregulation during the period. Interestingly, Tet2 showed downregulation in the first four days of being in the culture and an upregulation during the following four days [169]. 

#### 4.2.2. N6-Methyladenosine (m^6^a) RNA Methylation

M^6^a, which may occur in several forms of RNA, which include the miRNAs, represents the most common internal RNA post-transcriptional alteration in the eukaryotic cells [170]. Adenosine methyltransferases (METTL3, METTL14, and WTAP) act as writers [171,172], m^6^a -binding proteins act as readers, and m^6^a demethylating enzymes (FTO and ALKBH5) [173,174] act as erasers, and these are the key regulators of this reversible alteration [175]. 

It has been shown in other research that a proper hypertrophy response in the cardiomyocytes requires the methyltransferase-like 3 (METTL3)-associated methylation of the mRNA on N6-adenosines as an alteration that is amplified in correspondence to excessive stimulation. The fact that increased m^6^a RNA methylation produces a compensatory cardiac enlargement and a reduced m^6^A level of it promotes dynamic myocardium reconfiguration and impairment highlights the importance of this unique stress reaction mechanism in the heart for maintaining proper normal heart function [176].

In another study, METTL3 showed no substantial alterations in the cardiac tissue while inducing the transverse aortic constriction in a mouse model, while the FTO and WTAP expressions were attenuated. Additionally, it was discovered that cardiac hypertrophy started to develop in the mouse model 4 weeks after the transverse aortic constriction, and that the amount of total RNA that had been modified with the m^6^a codon increased in the hypertrophic heart tissues [177]. 

In another comprehensive research study, it was suggested that METTL3 was elevated in mice after they gave birth, which is the opposite of the alterations in the proliferation of the cardiomyocytes. The METTL3-pri-miR-143-(miR-143)-Yap/Ctnnd1 axis aids in m^6^A alteration to promote cardiac healing after an MI. To clarify, METTL3/m^6^A methylation loss inhibits the development of pri-miR-143 and regulates its production which results in a lower miR-143-3p yield. The transcription of Yap and Ctnnd1 (the receptors of miR-143-3p) rises significantly, promoting the multiplication of the heart cells and an innate recovery after an MI [178].

In another study, it was shown that ALKBH5 was essential for the proliferation and renewal of the cardiac myocytes. The upregulation of ALKBH5 transcription by the adeno-associated virus type 9 (AAV9) enhanced the percentage of replicating cardiac cells, decreased scar diameters, and normalized cardiac function following infarction damage, whereas ALKBH5 knockout in mice significantly restricted the rate of cardiomyocyte propagation and healing [179].

A well-studied cluster of miRNAs in the human ESC is the miR302-367 cluster. It has been revealed that stem cell-associated upstream transcription factors regulate this cluster in human ESCs. Nanog, Oct3/4, Sox2, and Rex1alleviate this cluster promoter expression [180], and consequently, the miR-302a from this cluster inhibits Cyclin D1, which is a significant G1 phase regulator in the human embryonic stem cells [181].

### 4.3. ATP-Dependent Chromatin Remodeling Complexes

ATP-dependent chromatin remodelers (ACRs) impact the DNA’s accessibility. They work by using ATP to break or modify the histone-DNA interaction. In other words, by sliding, twisting, or looping the nucleosomes, the ATP-dependent chromatin remodelers may modify the DNA’s accessibility [182]. These remodelers come in four families, with the switching defective/sucrose nonfermenting family receiving the most attention in the formation of the cardiomyocytes [183]. Nkx2-5, Gata4, Tbx5, and Tbx20 are a few cardiac transcription factors that interact with the Brahma-related gene 1 (Brg1)/Brahma (Brm)-associated factor complex [184]. Brg1 has a variety of functions in the development of the cardiomyocytes, including the activation of myosin heavy chains in fetuses and the inhibition of myosin heavy chains in adult cardiomyocytes and Bmp10-stimulated cardiomyocyte proliferation [185]. Tbx5, Gata4, and the Brg1/Brm-related factor component, Baf60c, have been shown to enhance the conversion of non-cardiac mesoderm into the heart muscle [186].

### 4.4. Cardiac Mitochondrial Mutations

In humans, cardiomyocyte development and myocardial infarction are both associated with an altered mitochondrial function [187,188]. In a study, it was discovered that the mitochondria-related genes in the cardiac cells in comparison to the other organs had different levels of methylation [189]. Numerous important processes, including ATP-dependent chromatin remodeling, are engaged in the epigenetic control of heart failure [190]. The SWI/SNF (SWItch/Sucrose Non-Fermentable) complex, which is made up of BRM and BRG1, is an ATP-dependent chromatin remodeling complex in the heart cells [191,192].

According to the research, Brg1/Brm double-mutant mice demonstrated enhanced mitophagy and dysregulated mitochondrial splitting and merging, which resulted in shattered mitochondria and increased mitochondrial biogenesis. These results outlined a function for BRG1 and BRM in mitophagy, mitochondrial dynamics, and abundance regulation as an epigenetic process [193].

The functions of mitochondrial-derived radicals in regulating the epigenetic pattern and gene function in the heart were proposed by another study. The findings demonstrated that mitochondrial-induced oxidative stress in the heart compromises myocardial DNA methylation, alters the heart gene transcription, and promotes the pathological heart changes that are indicative of cardiomyopathy [194]. Another research study found that nuclear DNA methylation was altered by a cardiac mitochondrial polymerase malfunction. The data suggest that mitochondrial impairment may affect DNA methylation as an epigenetic indicator, which is crucial for embryonic development [195].

It is documented that the pyruvate dehydrogenase stimulation that is caused by dichloroacetate may lead to epigenetic modification in the myocardium. Furthermore, dichloroacetate-dependent histone acetylation is linked to the up-regulation (by 2.3%) of the transcription-related genes, and the dichloroacetate-dependent cardiac mitochondrial production of acetyl-CoA is used to produce hydroxybutyrate, which is an innate blocker of the type I HDACs [196].

**Table 1:** Heart regeneration epigenetic factors, their modifications, target and effects.

Epigenetic Factors		Function	Affected Gene	Effect(s) on Cardiac Tissue					Species	References
				Evolution	Physiology	Pathology	Anomaly	Unknown		
HAT										
	p300	Activation	αMHC	+					Mice	[74,197]
		Activation	αSA	+					Mice	
		Activation	GATA4	+					Mice	[71,72,73]
		Activation	Nkx2.5	+					Mice	
		Activation	Mef2c	+					Mice	
	KAT2B					+			Human	[198]
	Gcn5			+					Rat	[80]
	CBP			+					Mice	[78,79]
	MOF					+			Mice	[77]
HDAC	HDAC1	Activation	Hopx	+					Mice	[82,83]
		Activation	GATA4	+					Mice	
	HDAC2	Activation	Hopx	+					Mice	
		Activation	GATA4	+					Mice	
	HDAC3	Inhibition	Mef2			+			Mice	[88,89]
		Inhibition	Cdkn1a				+		Mice	[84]
		Inhibition	Cdkn1b				+		Mice	
		Inhibition	Cdkn1c				+		Mice	
		Inhibition	Cdkn2b				+		Mice	
		Inhibition	Cdkn2c				+		Mice	
		Inhibition	Tbx5	+		+			Mice	[87]
	HDAC4	Inhibition	Mef2			+			Mice	[88,89]
	HDAC5	Inhibition	Mef2			+	+		Mice	[90,91]
	HDAC9	Inhibition	Mef2			+	+		Mice	
	SIRT1	Deacetylation	p21	+					Mice	[199]
	SIRT1					+			Mice	
	SIRT6	Activation	Oct4	+					Mice	[109]
		Activation	Sox2	+					Mice	
		Activation	Nanog	+					Mice	
	PTIP	Activation	Kcnip2		+				Mice	[124]
	Smyd1	Activation	skNAC	+					Mice	[117,128,129]
		Activation	Hand2	+					Mice	
		Activation	Irx4	+					Mice	
	Smyd2	Activation	NCHMTs	+					Mice	[132]
	Smyd5	Activation	H4K20me3	+		+			Mice	[200]
	Wolf-WHSC1	Activation	Nkx2.5	+			+		Mice	[134]
	PRC2	Activation	Oct4	+					Human	[53]
		Activation	Sox2	+					Human	
		Activation	Nanog	+					Human	
		Activation	EZH1	+					Mice	[137,138,201] [138,139,202]
		Activation	EZH2	+					Mice	
		Activation	Eed	+					Mice	[136]
		Activation	RbAp46/48	+					Mice	
		Activation	Suz12	+					Mice	
	G9a			+			+		Mice	[146]
	GLP			+			+		Mice	
	Suv39h1			+					Mice	[144]
	DOT1L	Activation	Nkx2.5	+					Human	[202]
HDM	KDM4a	Activation	ANP			+			Mice	[89,156]
		Activation	BNP			+			Mice	
	KDM4D	Activation	H3K9me3	+					Mice	[203]
	UTX	Activation	ANP	+					Mice	[122]
		Activation	MLC2	+					Mice	
		Activation	a-CA	+					Mice	
		Activation	TFs	+					Mice	
		Activation	Nkx2.5	+					Mice	
		Activation	Tbx5	+					Mice	
		Activation	GATA4	+					Mice	
		Activation	SRF	+					Mice	
		Activation	Baf60c	+					Mice	
	JmjC	NA	NA	+					Mice	[152]
	NO66			+					Mice	[204]
	Jmjd6			+			+		Mice	[154]
	Jmjd2a	Activation	BNP				+		Mice Human	[205]
	Jarid2	Activation	Notch1	+					Mice	[205,206]
		Activation	Fn1	+					Mice	
	Jmjd3	Activation	β-MHC	+		+			Mice Rat	[207,208]
	PHF8	Activation	pmaip1	+					Mice	[209]

αMHC, α-myosin heavy chain; αSA, α-sarcomeric actin; ANP, atrial natriuretic peptide; Baf60c, Brg1-associated factor 60c; BNP, brain natriuretic peptide; CBP, cAMP response element binding protein; Cdkn, cyclin-dependent kinase; CHD, Congenital heart defects; Eed, Embryonic ectoderm development; Ezh, catalytic subunit enhancer of Zeste; Fn1, Fibronectin 1; Hand2, heart- and neural crest derivatives-expressed protein 2; HAT, histone acetyltransferases; HDAC, histone deacetylases; HDM, histone demethylase; HMT, histone methyltransferase; Hopx, homeodomain-only protein; H3K9me3, lysine 9 of histone H3; Irx4, iroquois homeobox 4; Kcnip2, Kv channel-interacting protein 2; Mef2, myocyte-specific enhancer factor 2; MOF, males absent on the first; NCHMT, Neonatal cardiomyocytes histone methyltransferase; PHF19, PHD finger protein 19; PHF8, homeo domain finger protein 8; PRC2, polycomb repressive complex 2; PTIP, PAX-interacting protein 1; RbAp46/48, retinoblastoma protein associated protein 46/48; skNAC, skeletal nascent polypeptide-associated complex alpha; SRF, serum response factor; Suz12, suppressor of Zeste 12; UTX, ubiquitously transcribed tetratricopeptide repeat, X chromosome.

## 5. MicroRNAs (miRNAs)

### 5.1. miRNAs and Cardiomyocyte Differentiation

The microRNAs (miRNAs) play a larger role in iPSC differentiation and reprogramming. MiRNAs, which are small non-coding RNAs, are well understood to play a vital part in a number of processes. They are created from both the intragenic and the intergenic areas, and they may affect things such as body growth and development, the pathogenesis of autoimmune illnesses, different malignancies, hypertension, and more factors [210,211,212,213].

Furthermore, their role in respecting the pluripotency state of the stem cells has been assessed. Dicer-null and DGCR8-null ES cells are two instances of cells that are lacking mature miRNAs [214,215,216]. During differentiation, the Dicer-null cells show a very minor drop in the Oct4 expression levels [214], while the DGCR8-null ES cells exhibit an impaired development [216,217]. Thus, they are considered to be unique platforms for studying the significance of the miRNAs in pluripotency.

Just 18 miRNA families account for around 90% of the miRNAs in the heart [218]. The cardiomyocytes possess the highest concentration of miR-1 (which is a microRNA that is particular to the muscles) [219]. It has been demonstrated that a serum response factor controls its production in the rodent heart, where the levels steadily rise with the myocyte development, and they are at a peak in the adult heart as opposed to the embryonic or the neonatal stages [220]. In other words, the neonatal hypertrophic development of the heart is linked to comparatively lower levels of miR-1. It was discovered that miR-1 decreases during the stimulation of hypertrophy, perhaps through a pathway that is reliant upon a serum response factor [221].

Although the miR-133 expression is less than that of miR-1, both miRs are derived from the same bicistronic gene. MiR-1 and miR-133 in the ESCs collaborate to promote mesoblast development in the embryonic stem cells. Later on in their growth, they carry out opposite roles: miR-1 encourages the mesoblast development into cardiomyocytes, whereas miR-133 prevents it [222].

The levels of miRNA-1 and miRNA-133a steadily rise as the heart develops. Additionally, the induced amplification of miRNA-133a inhibits myocardial development, whilst miRNA-1 promotes it. The overexpression of both of the miRNAs increases the mesodermal commitment. The reduced production of miRNA-1 and miRNA-133a shows that both of the miRNAs have the opposite effect on heart growth compared to when they are upregulated [223]. Furthermore, the data show miRNA-1 and miRNA-133 control the transcription of Kdm6A/B and Ezh2 [224]. The adjustment of the Fgf8 activation, which is controlled by Bmp2, is regulated by miRNA-130 and miRNA-133. This regulatory feedback is necessary for attaining initial cardiac differentiation [225].

In the development of cardiomyocytes, miRNA-128 appears to play a direct function. It was demonstrated that the loss of functionality of miRNA-128 did not affect the atrial myocytes. Interestingly, the ventricles of miR-128 KO zebrafish larvae became smaller. The important cardiac transcription factors had their expression levels differently altered, which reflected this misdirected cardiogenesis in vitro (Isl1, Sfrp5, Nkx2.5, Mef2c, Irx4, and Hcn4). By influencing the timing of the cardiac progenitor cell differentiation into different cardiomyocyte subtypes, it was shown that miR-128a performs a yet unidentified function during early heart development [226]. 

MiR-134 may regulate the proliferation of human cardiomyocyte progenitor cells, as evidenced by the discovery that Meis2 is a target of the miRNA. This suggests that miR-134 may be involved in cardiogenesis [227]. 

Other investigations suggested that the miR-222 activity performs protection against negative ventricular remodeling and cardiac dysfunction following an ischemia damage. Additionally, these benefits were linked to the suppression of cardiomyocyte death and a significant reduction in fibrosis long after the ischemia damage [228]. Delivering miR-222 in conjunction with three other miRNAs also enhanced the calcium absorption rate and the sarcomere orientation, and consequently, the resting voltage became more negative and the production of the proteins in favor of cardiomyocyte development rose [229].

MicroRNA-294 was shown to have a unique function in the cell cycle of heart cells and myocardial regeneration. After a myocardial damage, the temporary expression of miRNA-294 promotes pro-reparative alterations in the cardiac tissue along with the restart of the myocyte cell cycle, improving the cardiac shape and function. Along with an increased cardiac cell cycle rate, an enhanced vasculature, and a decreased stroke lesion volume in the myocardium following the trauma, miR-294 also increases the cell viability [230].

While the miR-31a-5p transcription in cardiac myocytes was substantially elevated on the tenth postnatal day when it was compared to that of the first postnatal day, it was unexpectedly shown that miR-31a-5p promoted cardiac growth, whereas miRNA-31a-5p suppression inhibited proliferation. Using intact neonatal rat ventricular cardiac cells, this miRNA is the first miRNA from a subsequent myocardial development study to be shown to enhance cell proliferation [231].

In terms of the suppressive effect side of the miRNAs on their targets which comprise a major part of their functions, the results show that miR-200c inhibits the development and maturation of the hESCs. First of all, it blocks the ion channel and the transcription factors translation when it is acting alone. Second, miR-200c regulates many gene programs that are linked to heart growth and function by regulating the cardiac transcription factors [232].

Since MiR-375 is produced at the beginning of the cardiac development process, its upregulation greatly increases the mortality and deformity rates. Additionally, there was a considerable downregulation in the expression levels of the genes linked to heart growth. The investigations have revealed that the excessive transcription of miR-375 may result in deficiencies in the morphology of several organs and abnormalities of the downstream NOTCH signaling pathway (which shows potential as being a CHD therapeutic target and a diagnostic marker) [233].

In a zebrafish model, it was shown that miR-25 targets the cell cycle regulator FBXW7, and that miR-25 overexpression encourages cardiomyocyte multiplication by suppressing FBXW7. As a result, miR-25 may be a cutting-edge agent for heart regeneration [234]. In the research that was conducted by Yu et al., it was shown that miR-23 overexpression dramatically increased the proliferation of the AC16 cells and decreased cell apoptosis. According to these findings, miR-23 decreased the cardiomyocyte death rate, while it promoted proliferation by targeting TGF-1 [235].

### 5.2. miRNAs and Congenital Heart Defects

According to the strong findings, the miRNAs may be aberrantly expressed during the development of congenital heart defects (CHD), which are one of the most prevalent inherent abnormalities in newborns and children [236]. The regulation of cardiac morphogenesis, conductivities, and the cell-cycle adjustment are all influenced by the disruption in the miRNA synthesis [237].

For instance, DNA methyltransferases and the miR-29b-3p levels were negatively correlated in the CHD patients [238]. For cardiac cell proliferation and cardiac abnormalities, certain miRNAs (hsa-miR-221-3p, hsa-miR-218-5p, and hsa-miR-873-5p) have been described in [239]. The data support the idea that the CHD patients’ illness state is caused by a changed miRNA expression. In another study, it was revealed that hsa-miR-148a and BCL2L11 are linked to cardiomyocyte apoptosis and Hsa-miR-148a might affect BCL2L11 [240]. Recent findings have shown that miR-153-3p targets βII spectrin, which has a negative effect on myocardial development [241].

Researchers have found that improving the myocardial index, histological alterations in the myocardial tissue, and the apoptosis level by up-regulating miR-30c-5p or down-regulating BCL2L11 might have a molecular therapeutic impact on people with congenital heart defects [242]. In another related research, it was discovered that inhibiting miR-375-3p targeted and elevated FOXP1 and Bcl2l2 to suppress an hypoxia-evoked death of the cardiomyocytes. These findings may point to maternal serum, miR375-3p, as a promising biomarker for early fetal CHD identification [243]. All of these discoveries could pave the way for the future use of these miRNAs as diagnostics and targets for the detection and management of cardiac disorders.

**Figure 2:**
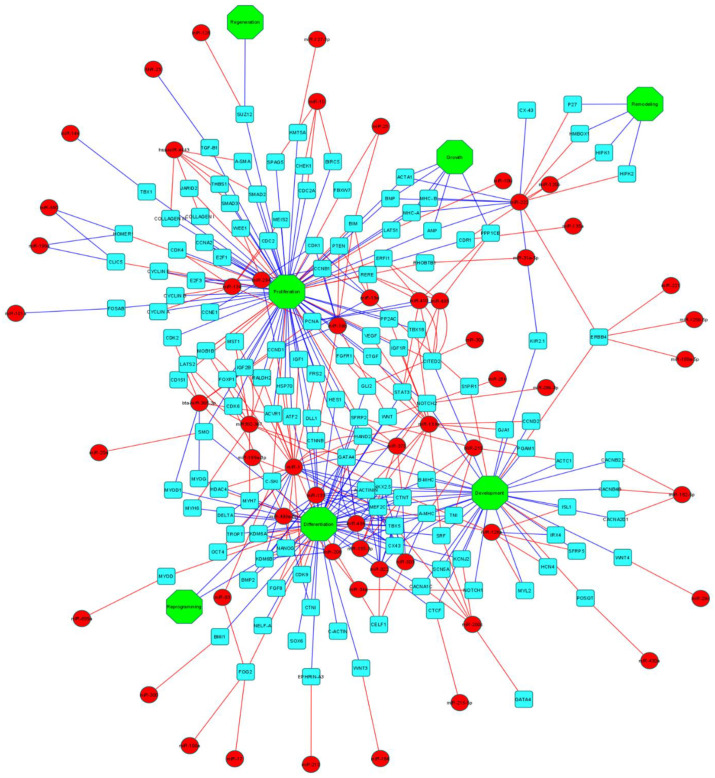
**Epigenetic roles of miRNAs in cardiac-specific gene expression.** Various miRNA subtypes (green circle) role in the inhibition (red arrows) and activation (dark blue arrows) of specific transcription factors (light blue triangle), which are responsible for differentiation, proliferation, development, growth, remodeling, reprogramming, and regeneration of cardiomyocytes (green octagon). (Figure 2 was generated using Cytoscape application—cytoscape.org (accessed on 28 September 2022)).

Various functions of the related miRNAs involved in the differentiation, proliferation, development, and possible reprogramming of the cardiomyocytes are summarized in Figure 2 and Table 2.

## 6. Conclusions

In this review, we have discussed numerous strategies for applying in iPSC and ESC-based future investigations that focus on cardiomyocyte regeneration. These approaches consist of various post-transcriptional modifications involving the DNA, RNAs, histone modifications, the ATP-dependent chromatin remodeling complexes, and mutations in the mitochondria of the cardiac cells. These studies together certainly provide a broad range of elements that should be taken into consideration while one is performing heart regeneration studies in the future. 

There are other methods outside of this review for pursuing cardiac cell regeneration as well. For instance, an approach for reactivating the ability of the cardiomyocytes toward regeneration is the utilization of extracellular vesicles (EVs), which also deliver miRNAs as part of their cargo. In recent research, it was shown that human and mouse epicardial cells secrete EVs, and that these EVs may stimulate the proliferation of cardiac myocytes in vitro. The effective epicardial EV absorption by heart cells was shown to encourage cell cycle activation [288]. Another study suggested that miRNA-21-loaded EVs persistently prevented cell death, and they have significantly enhanced the cardiovascular output in an animal model of preclinical MI by injecting produced EVs as well [289].

Various types of cells have been discovered to release the EVs that encourage the cardiomyocyte cell cycle activity as well [290,291]. Other studies have supported the hypothesis that turning the attention away from the iPSCs and the ESCs and onto their secreted EVs may be the answer to the ever-increasing problems that are associated with dealing with stem cells [292]. 

Another intriguing approach was the utilization of exosome-derived miRNA-19a/19b and the MSCs, simultaneously, which significantly improved the restoration of the cardiovascular output and decreased the heart fibrosis in an infarction model [293]. A final solution has not been advised by any of these methods, even though they have all shown us extremely encouraging results. Therefore, further preliminary studies must be conducted to evaluate the efficacy of all of these treatments and their effects on the various cell signaling pathways in the heart muscle in the long run.

The discrepancies between the complicated in vivo environments and the in vitro experiments could be effectively reduced by using microfluidic technology as well [294]. For instance, tissue engineering microfluidic devices which can be utilized while executing electrical stimulation [295], mechanical stimulations (such as a cell stretching device with special substrates or by providing shear stress) [296,297], biochemical stimulation [298], physical factors, and structural stimulations [5] have been suggested to simulate the natural heart environment. In a study, the researchers proposed a system with the capability to deliver every stimulation separately or to integrate three separate impulses to examine the collaboration of multiple stimuli, which would better simulate the complicated in vivo environment [299].

Biochemical reagents for the differentiation of the MSCs are utilized commonly as well. For instance, Azacytidine (5-AZA) has been recognized as a biochemical reagent for reprogramming cells toward contractile striated muscle cells since 1979 [300]. It is still in use today, with new approaches having been taken for the better differentiation of the MSCs into cardiomyocytes. To differentiate the hMSCs, pharmacological agents such as the vascular endothelial growth factor (VEGF) and 5-AZA were utilized in one study by using a microfluidic system, which is capable of the precise regulation of the media flow, its timing, the pattern of the reagent supply, and the incubation period of the cultured cells. The authors have announced promising results using this combined approach as well [301].

Another study supported the idea that TGF-β1 is non-toxic to cells, effective, and may be a better inducer of stem cell to cardiomyocyte differentiation than 5-AZA [302] due to the effect of it on recognized TGF/BMP signaling pathway versus that of 5-AZA, which is a demethylating agent with poorly characterized mechanism [303,304].

Collectively, it should be considered that the cellular micro-environment is composed of an intricate network of stem cells, satellite cells, bioactive substances, and the extracellular matrix. The microenvironment is influenced by protein adhesives (such as laminin, fibronectin, and vitronectin), fibrous structural proteins (such as elastin and collagen), polysaccharides in the form of glycosaminoglycan, and proteoglycan additionally. All of these variables should be precisely taken into account while one is conducting stem cell differentiation experiments [301,305].

## Figures and Tables

**Table 2:** MicroRNAs involved in heart regeneration, their target, and their effect.

MicroRNAs	Effect(s) on Gene	Affected Gene	Gene Function	References
miR-1	Activation	DELTA	Differentiation	[221,223,224,244,245]
	Inhibition	CDK9	Inhibition of differentiation	
	Inhibition	GATA4	Proliferation	
	Inhibition	HAND2	Proliferation	
	Inhibition	HSP70	Proliferation	
	Inhibition	RALDH2	Proliferation	
	Inhibition	IGF2B	Proliferation	
	Inhibition	IGF1	Proliferation	
	Inhibition	FRS2	Proliferation	
	Inhibition	FOXP1	Proliferation	
	Inhibition	HAND2	Inhibition of differentiation	
	Inhibition	HDAC4	Inhibition of differentiation	
	Activation	NKX2.5	Differentiation	
	Inhibition	CCND1	Proliferation	
	Inhibition	NANOG	Inhibition of differentiation	
	Inhibition	OCT4	Inhibition of differentiation	
	Activation	TROPT	Differentiation	
	Activation	A-ACTININ	Differentiation	
	Activation	KDM6A	Differentiation	
	Activation	KDM6B	Differentiation	
	Activation	TBX5	Differentiation	
	Activation	MEF2C	Differentiation	
	Activation	GATA4	Differentiation	
miR-10b	Inhibition	LATS1	Inhibition of proliferation	[246]
miR-15	Inhibition	CHEK1	Proliferation	[247]
	Inhibition	CDC2A	Proliferation	
	Inhibition	BIRC5	Proliferation	
	Inhibition	SPAG5	Proliferation	
miR-17	Inhibition	FOG2	Inhibition of differentiation	[248]
miR-19a	Inhibition	S1PR1	Inhibition of development	[249,250]
	Inhibition	BIM	Inhibition of proliferation	
	Inhibition	PTEN	Inhibition of proliferation	
	Activation	CCNB1	Proliferation	
	Activation	CCND1	Proliferation	
	Activation	CDK1	Proliferation	
miR-19b	Inhibition	WNT	Differentiation	[228,250]
	Inhibition	CTNNB	Differentiation	
	Inhibition	PTEN	Inhibition of proliferation	
	Inhibition	BIM	Inhibition of proliferation	
	Activation	CCNB1	Proliferation	
	Activation	CCND1	Proliferation	
	Activation	CDK1	Proliferation	
miR-20a	Inhibition	SMO	Proliferation Differentiation	[251]
MiR-23	Activation	TGF-Β1	Proliferation	[235]
miR-25	Inhibition	FBXW7	Inhibition of proliferation	[234,252]
	Inhibition	BIM	Inhibition of proliferation	
miR-26b	Inhibition	WNT	Differentiation	[253]
miR-29c	Inhibition	WNT4	Development	[254]
miR-29b-3p	Inhibition	NOTCH2	Proliferation	[255]
			Development	
miR-30c	Inhibition	GLI2	Inhibition of proliferation	[256]
			Differentiation	
miR-31a-5p	Inhibition	RHOBTB1	Inhibition of proliferation	[231]
miR-34a	Inhibition	C-SKI	Inhibition of proliferation	[257,258]
	Inhibition	NOTCH1	Development	
miR-93	Inhibition	FOG2	Inhibition of differentiation	[248,257]
	Inhibition	C-SKI	Inhibition of proliferation	
miR-101a	Activation	FOSAB	Proliferation	[259]
miR-106a	Inhibition	FOG2	Inhibition of differentiation	[248]
miR-125b-5p	Inhibition	ERBB4	Inhibition of development	[229]
mir-127-3p	Inhibition	KMT5A	Proliferation	[260]
miR-128	Inhibition	SUZ12	Proliferation	[261]
			Regeneration	
miR-128a	Inhibition	ISL1	Development	[226]
	Inhibition	SFRP5	Development	
	Inhibition	HCN4	Development	
	Inhibition	NKX2.5	Development	
	Inhibition	MEF2C	Development	
	Inhibition	MYL2	Development	
	Activation	IRX4	Inhibition of development	
miR-133	Activation	BMP2	Differentiation	[224,225]
	Activation	GATA4	Differentiation	
	Activation	NKX2.5	Differentiation	
	Inhibition	FGF8	Inhibition of differentiation	
	Activation	KDM6A	Differentiation	
	Activation	KDM6B	Differentiation	
	Activation	TBX5	Differentiation	
	Activation	MEF2C	Differentiation	
	Activation	CX43	Differentiation	
	Activation	CTNT	Differentiation	
miR-133a	Inhibition	NKX2.5	Development	[223,244,247,262,263,264]
	Inhibition	PGAM1	Development	
	Inhibition	GJA1	Development	
	Inhibition	SRF	Development	
	Inhibition	CCND2	Development	
	Inhibition	IGF1R	Proliferation	
	Inhibition	HAND2	Development	
	Inhibition	A-ACTININ	Development	
	Inhibition	CTNT	Development	
	Inhibition	VEGF	Proliferation	
	Inhibition	CTGF	Proliferation	
	Inhibition	FGFR1	Proliferation	
	Inhibition	PP2AC	Proliferation	
	Inhibition	TBX18	Proliferation	
miR-134	Inhibition	MEIS2	Proliferation	[227]
	Inhibition	CYCLIN A	Proliferation	
	Inhibition	CYCLIN B	Proliferation	
	Inhibition	CYCLIN E	Proliferation	
	Inhibition	CDC2	Proliferation	
	Inhibition	CDK4	Proliferation	
	Inhibition	PCNA	Proliferation	
miR-135a	Inhibition	CDR1	Proliferation	[265]
miR-135b	Inhibition	CDR1	Proliferation	[265]
miR-144	Activation	TBX1	Proliferation	[266]
miR-155-3p	Inhibition	MEF2C	Differentiation	[267]
	Inhibition	CTNT	Development	
	Inhibition	GATA4	Development	
	Inhibition	NKX2.5	Development	
miR-182-5p	Inhibition	CACNB2.2	Development	[268]
	Inhibition	CACNB4B	Development	
	Inhibition	CACNA2D1	Development	
miR-184	Inhibition	WNT3	Differentiation	[269]
miR-199a	Activation	CLIC5	Inhibition of proliferation	[247]
	Activation	HOMER1	Inhibition of proliferation	
miR-199a-3p	Inhibition	CD151	Inhibition of proliferation	[270]
	Inhibition	MEF2C	Differentiation	[271]
miR-199a-5p	Inhibition	ERBB4	Inhibition of development	[229]
miR -200c	Inhibition	GATA4	Differentiation	[232]
			Development	
	Inhibition	SRF	Differentiation	
			Development	
	Inhibition	TBX5	Differentiation	
			Development	
	Inhibition	CACNA1C	Differentiation	
			Development	
	Inhibition	KCNJ2	Differentiation	
			Development	
	Inhibition	SCN5A	Differentiation	
			Development	
miR-204	Inhibition	JARID2	Inhibition of proliferation	[272]
miR302-367	Inhibition	MST1	Inhibition of proliferation	[273]
	Inhibition	LATS2	Inhibition of proliferation	
	Inhibition	MOB1B	Inhibition of proliferation	
	Inhibition	CCND1	Proliferation	
	Inhibition	GATA4	Differentiation	
	Inhibition	NKX2.5	Development	
	Inhibition	MYH6	Differentiation	
	Inhibition	MYH7	Differentiation	
bta-miR-365-3p	Inhibition	ACVR1	Inhibition of differentiation	[274]
			Proliferation	
	Inhibition	CCND1	Proliferation	
	Inhibition	CDK2	Proliferation	
	Inhibition	PCNA	Proliferation	
	Activation	MYOD1	Differentiation	
	Activation	MYOG	Differentiation	
hsa-miR-4443	Inhibition	THBS1	Proliferation	[275]
	Inhibition	COLLAGEN I	Proliferation	
	Inhibition	COLLAGEN III	Proliferation	
	Inhibition	SMAD2	Proliferation	
	Inhibition	SMAD3	Proliferation	
	Inhibition	A-SMA	Proliferation	
miR-208	Activation	KDM6A	Differentiation	[224]
	Activation	KDM6B	Differentiation	
	Activation	TBX5	Differentiation	
	Activation	MEF2C	Differentiation	
	Activation	GATA4	Differentiation	
miR-210	Inhibition	EPHRIN-A3	Differentiation	[276,277]
miR-215-5p	Inhibition	CTCF	Development	[278]
			Differentiation	
MiR218	Inhibition	SFRP2	Inhibition of proliferation	[279]
			Differentiation	
	Inhibition	A-MHC	Development	
			Differentiation	
	Inhibition	Β-MHC	Development	
	Inhibition	ACTC1	Development	
	Inhibition	TNI	Differentiation	
miR-221	Inhibition	ERBB4	Inhibition of development	[229]
miR-222	Activation	ANP	Proliferation	[228,229]
			Growth	
	Activation	BNP	Proliferation	
			Growth	
	Activation	ACTA1	Proliferation	
			Growth	
	Activation	MHC-A	ProliferationGrowth	
	Activation	MHC- Β	Proliferation	
			Growth	
	Activation	CX-43	Development	
	Activation	KIR2.1	Development	
	Inhibition	ERBB4	Inhibition of development	
	Inhibition	HIPK1	Remodeling	
	Inhibition	HIPK2	Remodeling	
	Inhibition	HMBOX1	Remodeling	
	Inhibition	P27	Remodeling	
miR-294	Inhibition	WEE1	Inhibition of proliferation	[230]
	Activation	CCNB1	Proliferation	
	Activation	CCND1	Proliferation	
	Activation	CCNE1	Proliferation	
	Activation	CCNA2	Proliferation	
	Activation	CDK1	Proliferation	
	Activation	E2F1	Proliferation	
	Activation	E2F3	Proliferation	
miR-300	Inhibition	BMI1	Differentiation	[280]
miR302-367	Inhibition	MST1	Inhibition of proliferation	[273]
	Inhibition	MOB1B	Inhibition of proliferation	
	Inhibition	LATS2	Inhibition of proliferation	
miR-322	Inhibition	CELF1	Inhibition of differentiation	[281]
	Activation	TBX5	Differentiation	
	Activation	MEF2C	Differentiation	
	Activation	NKX2.5	Differentiation	
	Activation	A-MHC	Differentiation	
	Activation	A-ACTININ	Differentiation	
	Activation	CTNT	Differentiation	
miR-375	Inhibition	NOTCH2	Differentiation	[233,282]
			Proliferation	
			Development	
	Inhibition	DLL1	Differentiation	
			Proliferation	
	Inhibition	HES1	Differentiation	
			Proliferation	
	Inhibition	CTNT	Differentiation	
	Inhibition	NKX2.5	Differentiation	
	Inhibition	GATA4	Differentiation	
miR-410	Inhibition	CITED2	Inhibition of proliferation	[283]
	Activation	PCNA	Proliferation	
	Inhibition	CITED2	Development	
	Inhibition	ERFI1	Inhibition of proliferation	
	Inhibition	PPP1CB	Growth	
	Inhibition	RERE	Inhibition of proliferation	
	Inhibition	STAT3	Differentiation	
miR-430a	Inhibition	POSQT	Inhibition of development	[284]
miR-495	Inhibition	CITED2	Inhibition of proliferation	[283]
	Activation	PCNA	Proliferation	
	Inhibition	CITED2	Development	
	Inhibition	ERFI1	Inhibition of proliferation	
	Inhibition	PPP1CB	Growth	
	Inhibition	RERE	Inhibition of proliferation	
	Inhibition	STAT3	Differentiation	
miR-499	Inhibition	KDM6A	Reprogramming	[224]
	Inhibition	KDM6B	Reprogramming	
	Activation	TBX5	Differentiation	
	Activation	MEF2C	Differentiation	
	Activation	GATA4	Differentiation	
	Activation	CX43	Differentiation	[285]
	Activation	CTNT	Differentiation	
	Activation	NKX2.5	Differentiation	
	Activation	GATA4	Differentiation	
miR -499a-5p	Activation	A-ACTININ	Differentiation	[286,287]
	Activation	CTNI	Differentiation	
	Inhibition	CDK6	Proliferation	
miR-503	Inhibition	CELF1	Inhibition of differentiation	[281]
	Activation	TBX5	Differentiation	
	Activation	MEF2C	Differentiation	
	Activation	NKX2.5	Differentiation	
	Activation	A-ACTININ	Differentiation	
	Activation	A-MHC	Differentiation	
	Activation	CTNT	Differentiation	
miR-590	Activation	CLIC5	Inhibition of proliferation	[247]
	Activation	HOMER1	Inhibition of proliferation	
miR-699a	Inhibition	MYOD	Inhibition of differentiation	

## Data Availability

The data used to support the findings of this study are included within the article.

## Abbreviations

MSCs	Mesenchymal stem cells
hMSCs	Human mesenchymal stem cells
ESCs	Embryonic stem cells
iPSCs	Induced pluripotent stem cells
miRNAs	MicroRNAs
siRNA	Small interfering RNA
piRNA	Piwi-interacting RNA
HATs	Histone acetyltransferases
HDACs	Histone deacetylases
MOF	Males absent on the first
SIRTs	Sirtuins
SIRT1	Sirtuin 1
SIRT2	Sirtuin 2
SIRT3	Sirtuin 3
SIRT4	Sirtuin 4
SIRT5	Sirtuin 5
SIRT6	Sirtuin 6
SIRT7	Sirtuin 7
PTIP	PAX-interacting protein 1
PRC2	Polycomb repressive complex 2
UTX	Ubiquitously transcribed tetratricopeptide repeat, X chromosome
CHD	Congenital heart defect
EVs	Extracellular vesicles
VEGF	vascular endothelial growth factor
5-AZA	5-azacytidine
